# Manufacture and Combustion Characteristics of Cellulose Flame-Retardant Plate through the Hot-Press Method

**DOI:** 10.3390/polym15244736

**Published:** 2023-12-18

**Authors:** Jeo Hwang, Dongin Park, Dongho Rie

**Affiliations:** 1 Graduate School of Safety Engineering, Incheon National University, Incheon 22012, Republic of Korea; hjojjang@inu.ac.kr; 2 Department of Safety Engineering, Incheon National University, Incheon 22012, Republic of Korea; sunydong@inu.ac.kr; 3 Fire Disaster Prevention Research Center, Incheon National University, Incheon 22012, Republic of Korea

**Keywords:** cellulose, hot press, building materials, cone-calorimeter, NES-713, fire safety

## Abstract

This study focuses on the increased risk of high heat release and asphyxiation (toxic gas poisoning) in the event of a fire involving polyurethane (PU)- and MDF-based building materials, which are commonly used in buildings. Among them, polyurethane (PU) building materials are very commonly used in buildings, except in Europe and some other countries, due to their excellent thermal insulation performance. Still, problems of short-term heat release and the spread of toxic gases in the event of a fire continue to occur. To overcome these problems, researchers are actively working on introducing various flame retardants into building materials. Therefore, in this study, we produced a laboratory-sized (500 mm × 500 mm) plate-like flame-retardant board that can be utilized as a building material with a lower heat release rate and a lower toxicity index. The material was made by mixing expanded graphite and ceramic binder as flame retardants in a material that is formulated based on the cellulose of waste paper, replacing the existing building materials with a hot-press method. According to the ISO-5660-1 test on the heat release rate of the plate-like flame-retardant board, the Total Heat Release (THR) value was 2.9 (MJ/m^2^) for 10 min, showing an effect of reducing the THR value by 36.3 (MJ/m^2^) compared to the THR value of 39.2 (MJ/m^2^) of the specimen made using only paper. In addition, the toxicity index of the flame-retardant board was checked through the NES (Naval Engineering Standards)-713 test. As a result, the test specimen showed a toxicity index of 0.7, which is 2.4 lower than the toxicity index of 3.1 of MDF, which is utilized as a conventional building material. Based on the results of this study, the cellulose fire-retardant board showed the effect of reducing the heat release rate and toxicity index of building materials in a building fire, which reduces the risk of rapid heat spread and smoke toxicity. This has the potential to improve the evacuation time (A-SET) of evacuees in fires. It is also important to show that recycling waste paper and utilizing it as the main material for building materials can be an alternative in terms of sustainable development.

## 1. Introduction

According to the National Fire Data System (NFDS) of the Republic of Korea (ROK), out of 197,480 total fires from 2018 to 2022, there were 128,390 fires in building structures, accounting for 65% of the total. In addition, the total number of human casualties as a result of fires was 10,047, with 1354 deaths [1]. The most common cause of casualty of all fires was burns, with 1065 cases in 2022, an increase of more than 8% from 986 cases in 2018. In addition, there was an increase of 158 cases of smoke and toxic gas inhalation, from 685 cases in 2018 to 843 cases in 2022, showing a trend of increasing heat and smoke hazards [2].

The fire resistance performance of building materials has gained global attention because it is a critical factor in fire response and evacuation. Variables such as fire resistance, durability, smoke emissions, and toxicological hazards of building materials are critical in increasing the likelihood of escape and survival for occupants of residential buildings [3].

One of the most commonly used building materials is polyurethane (PU), which is molded into a foam shape—PU foams (PUFs) [4]. PUFs are porous polymers and are often used for thermal insulation in building materials [5]. Although these PUFs have low thermal conductivity, which makes them excellent for thermal insulation, PUFs are flammable and can contribute to the spread of fire. To compensate for these shortcomings, research is underway to introduce various types of flame retardants [6]. In addition, the manufacturing method based on chemical foaming has harmful effects on the environment and human health, which has become a global issue [7,8]. These issues have led to the globalization of building materials.

Due to these issues, research on basic materials for building materials is underway worldwide. Among them, studies based on cellulose are attracting attention.

In a study by Youngjoo Kim et al., samples of building materials were made using expanded graphite and magnesium hydroxide based on cellulose extracted from waste paper. The basic data were collected based on the ISO 5660-1 [9] test for the samples. The results of the FDS simulation based on the amount of oxygen, carbon monoxide, and carbon dioxide showed a fractional effective dose (FED) value that met the life safety standards of the National Fire Safety Codes 203 and did not exceed a maximum of 0.001. In addition, the cellulose-based building materials reduced the amount of smoke generated, thereby improving the escape time of occupants [10].

In a study by Sun, J. et al., a blend of cellulose, aerogel, and montmorillonite showed higher Total Heat Release (THR) values during the first 62 s of the test compared to the results of the cone calorimeter test for a blend of cellulose and aerogel, but the THR values were found to be lower after the end of the test. Furthermore, the addition of ammonium hydrogen phosphate resulted in a decrease of 73.33% in the maximum Peak Heat Release Rate (PHRR) value and 45.9% in the THR value compared to the cellulose–aerogel blended sample. This showed that the flame retardancy improved as the amount of added ammonium hydrogen phosphate increased [11].

Manufacturing methods and techniques for additive materials are also being studied. In a study by Rawers, J. et al., new materials were prepared by compressing Fe-Al and Fe-C powders using a hot-press method, and the results showed that the microstructural changes contributed to the strength improvement [12]. Candan, Z. et al. showed that, when paulownia boards were thermally compressed using an experimental hot press, the moisture content decreased and the density and hardness increased, suggesting that hot-press manufacturing is a viable alternative method for producing high-performance wood with an environmentally friendly approach [13]. Han, X. et al. successfully fabricated wood-based materials with excellent physical and mechanical properties and outstanding flame retardancy through chemical pretreatment, flame-retardant infusion, and hot-pressing processes. The tensile strength, Young’s modulus, and toughness were found to be 175.6 MPa, 1.2 GPa, and 22.9 MJ m^−3^, respectively; these values are significantly better than those of natural poplar wood. The flame retardancy test showed that the PFBS impregnation modification increased the flame retardancy of poplar wood, which greatly improved its practical safety [14].

A study by Wenyu Wu Klingler et al. presented various methods and directions for the sustainable reuse of polymeric materials; this is necessary in light of the increasing use of polymeric materials as flame-retardant materials in the fire safety field. The chemical properties were categorized into esters (carboxylic and phosphate esters), sulfur-containing linkages, nitrogen-containing structures, etc.; the researchers emphasized the use of bio-based raw materials. This showed the need for future research to consider the fire safety of flame-retardant materials, their repressibility, and recycling, as well as the current status of various research programs [15].

In addition, according to a study by Liang et al., the toxicity index of organic foam, polyurethane, and polyethylene building materials was evaluated to be higher than 10, indicating a high risk of smoke toxicity. Therefore, it was evaluated that non-combustible materials should be constructed along with building materials to ensure safety against smoke toxicity poisoning caused by fire [16].

Therefore, this study recognized the need for sustainable bio-based flame-retardant building materials and focused on ensuring fire safety through the use of flame-retardant materials, as shown in previous studies. Therefore, for the basic research of building materials with sustainable fire safety, specimens of building materials based on waste paper were manufactured and evaluated. Waste paper and flame-retardant additives were mixed and hot-pressed to produce experiment-sized boards. The specimens were evaluated for fire safety. The thermal properties of the specimens were tested according to ISO-5660-1, and the toxicity evaluation utilized the method of NES-713. This research aims to address the need for sustainable bio-based flame-retardant building materials and enhance fire safety in construction.

## 2. Method and Materials

### 2.1. Hot-Press Method: Overview

**Figure 1:**
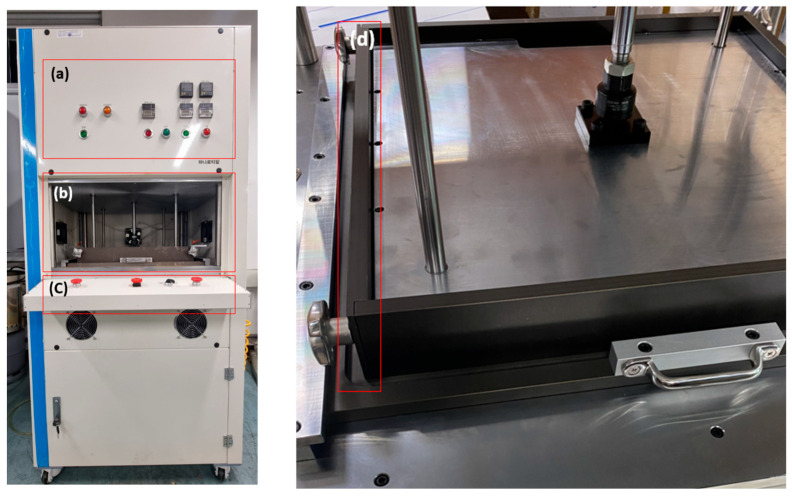
Laboratory-scale hot-press equipment. (**a**) Process indicators, (**b**) hot-press zone, (**c**) control panel, (**d**) hot-press sidewalls.

The structure of the hot press used to produce the specimens in this experiment is shown in Figure 1. The laboratory hot press has a structure in which the temperatures od the upper and lower plates can be individually adjusted up to a temperature of 150 degrees, and the upper plate rises and falls. In addition, the side walls can be assembled to facilitate the removal of plate-like specimens after they are produced. This function can be used to prevent damage to the flame-retardant board which can happen when removing it from the press without sufficient moisture release from the bottom plate. The PLC controls the operation at the temperature set by the researcher, and the temperature of the top and bottom plates are individually adjusted. For the safety of the researcher, the control panel uses an ambidextrous operation to prevent pinching and burning the researcher’s body. For the effective discharge of steam during the process, a steam-release line was constructed on the top plate. The kneaded composites were placed in the laboratory’s hot press, where the temperature was increased from 90 to 100 for the first 30 min to provide pressure; the specimen was heated for 1 h. The resulting specimens were dried in a desiccator for 24 h before being cut to size and used as experimental specimens.

### 2.2. Plate-like Flame-Retardant Board Specimen Materials

For the waste paper, corrugated cardboard was purchased from a local recycling center in Incheon, Republic of Korea. This was used as the source of wastepaper in this study. Expanded graphite and ceramic binder were used as flame-retardant materials to reduce the heat-emission rate and the toxicity index of the plate-shaped flame-retardant board. Expanded graphite has been used in expandable flame-retardant systems to provide successful flame-retardant properties compared to other materials, such as magnesium hydroxide (Mg(OH)_2_) and titanium dioxide (TiO_2_) [17]. The ceramic binder used in this experiment was ‘FP-100′, sourced from Sunjin Chemical Co., Seoul, Republic of Korea. The ceramic binder is composed of 48% MgO, 17% SiO_2_, 14.6% Al_2_O_3,_ and other components. In addition, expanded graphite is a 100-mesh product sold by Samjung C&E, Gyeongsan City, Republic of Korea.

### 2.3. Basic Dough for Flame-Retardant Boards

The pulping was performed using a pulper with a mixture of waste paper and water. The pulper was operated at a motor speed of 360 RPM for 60 min, with a maximum capacity of 16 L.

After the pulping process was completed, the primary dehydration process was performed. The basic dough was prepared by mixing cellulose flame-retardant materials with a certain amount of moisture removed. The base dough was made into a laboratory-scale plate-shaped building material through a hot-pressing process. Additives were applied at a rate of 100 Wt.% in accordance with the weight of the waste paper. The units were expressed as Weight % (Wt.%). The content of ceramic binder was added at 30 Wt.% and 40 Wt.%. In addition, expanded graphite proportions of 40 Wt.% and 50 Wt.% were mixed according to the matrix in Table 1. To check the fire properties of the cellulose-only board, specimens without additives were also prepared as controls.

### 2.4. The Hot-Press Specimen-Making Process

First, the hot-press machine was preheated to a temperature of 90 °C. Then, the top and bottom plates were covered with paper foil to prevent the plate-like building materials from sticking to the machine after the process. The base dough was then applied to the bottom plate.

When the second application was completed, the top plate was moved towards the bottom plate through operation of the two-handed button to press the weight of the top plate through pneumatic pressure. The pneumatic pressure was transmitted through the cylinder. The working pressure of the cylinder used for pneumatic pressure was 9.9 Kgf/cm^3^, and the internal cross-sectional area of the cylinder was 30.95 cm^3^. Therefore, the pneumatic pressure of the hot press was pressed with a force of about 3 KN. The temperature was then raised to 100 °C for 30 min to maintain a constant temperature. After that, pressing was carried out for 60 min while maintaining a temperature of 100 °C.

**Figure 2:**
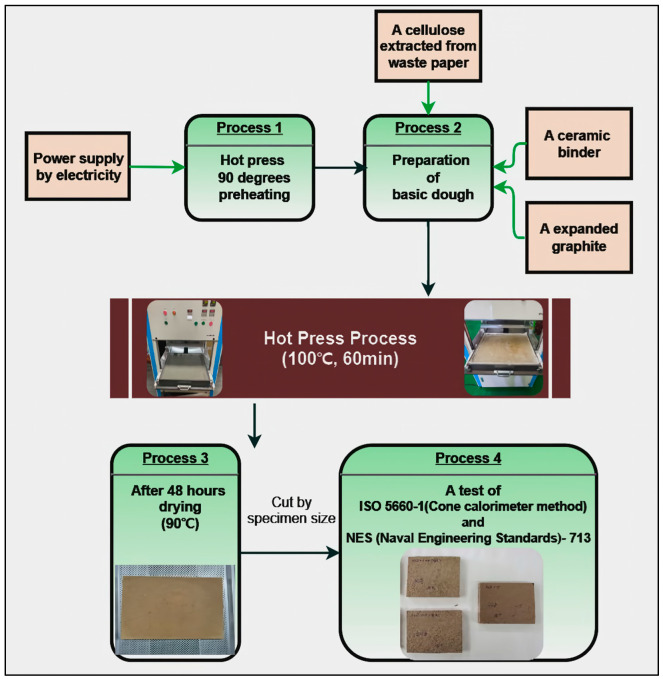
A test piece hot-press production flow chart.

Finally, once the process was complete, the top plate was pneumatically lifted upward. The bottom plate was then positioned in front of the researcher to remove the sidewalls, and the plate-like specimen was transferred to a drying plate for post-process drying. The plate was then placed in a dryer to dry at a temperature of 90 °C for 48 h. Figure 2 shows a process flow chart of the hot-pressing process. The plate-like flame-retardant board was cut at three random points to meet the specifications of the test specimens for ISO-5660-1 and NES-713 tests.

### 2.5. NES-713 Experimental Materials

In this experiment, among the products distributed and used as building interiors, PF Board (inner core only), compressed polystyrene foam, MDF, and hot-pressed panel board produced in-house using waste paper were used as specimens. In addition, PF board, compressed polystyrene foam, and MDF were purchased and used as random materials distributed in the market. The appearance and components of the specimens are shown in Table 2. The specimens were cut to a weight of 1 ± 0.2 g.

## 3. Fire and Toxicity Assessment

### 3.1. Description of ISO 5660-1 Test

**Figure 3:**
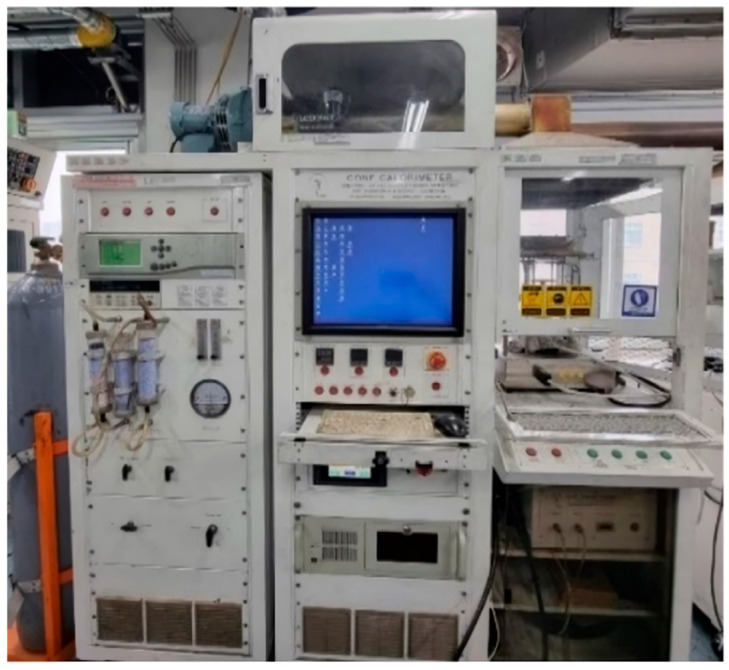
A ISO 5660-1 experimental device (cone calorimeter).

Cone calorimeter testers are often used in small-scale experiments to test the fire behavior of building materials. Figure 3 shows the cone calorimeter tester used in this experiment. The International Organization for Standardization has adopted the cone calorimeter tester as a standard for measuring the Heat Release Rate (HRR) of the test materials. The standard is specified in ISO-5660-1. Huggett, C., confirmed that the heat released from building materials is proportional to the oxygen consumed during combustion. He also confirmed that most fuels produce about 13.1 MJ of energy per kilogram of oxygen, based on experiments with different types of test materials [18]. Based on these results, the amount of oxygen consumed is measured to collect data such as those for HRR and THR, which are important parameters in fire experiments and fire risk assessment.

The experiments were conducted as follows: The specimen dimensions required by the standard are 100 × 100 mm². The specimen is wrapped in aluminum foil, which is joined to a holder so that only the top is exposed to the heat emitted by the heating element in the cone calorimeter tester. A spark igniter is placed over the specimen surface and baseline data are collected for 60 s before the actual combustion test begins. The experimental data are then measured relative to this baseline [9].

Table 3 shows the classification of fire-resistant materials according to the fire characteristics of building materials in Republic of Korea and Japan. In the case of the Republic of Korea, it is classified as a Semi-Non-Combustible Material and Fire-Retardant Material when the THR does not exceed 8 MJ [19]. In the case of Japan, it is classified as flame-retardant class 1 (non-combustible), flame-retardant class 2 (semi-combustible), and flame-retardant class 3 (flame retardant) when the THR does not exceed 8 MJ [20]. Both countries use ISO 5660-1 for the evaluation method. In addition, in the United States, the National Fire Code (NFC) of the National Fire Protection Association (NFPA) has established the Building Code, IBC, which is used as a code for the application of fire safety standards in buildings. According to NFC’s NFPA255 (Standard Test Method for Surface Combustion Characteristics of Building Materials), the following metrics are used to are used to classify the classes into A, B, and C: the flame spread index, FIGRA (Fire Growth Rate Index), which is the maximum value of the index for the rate and time of heat release from the test object; the smoke growth rate index, SMGRA (Smoke Growth Rate Index), which is the maximum value for the rate and time of smoke production from the test object [21]. In this study, the flame-retardancy performance was compared and evaluated using Japanese flame-retardant ratings.

### 3.2. Description of NES 713 Test

NES (Naval Engineering Standards) 713 tests were conducted to determine the smoke toxicity of currently used building materials and specimens. NES 713 tests are conducted to determine the composition and emission of toxic gases in building materials. The methodology of the NES 713 toxicity test is based on the Naval Engineering Standard code [22]. The NES 713 test provides a relative toxicity index for concentrations that can cause death after 30 min of exposure to a total of 13 gases.

**Figure 4:**
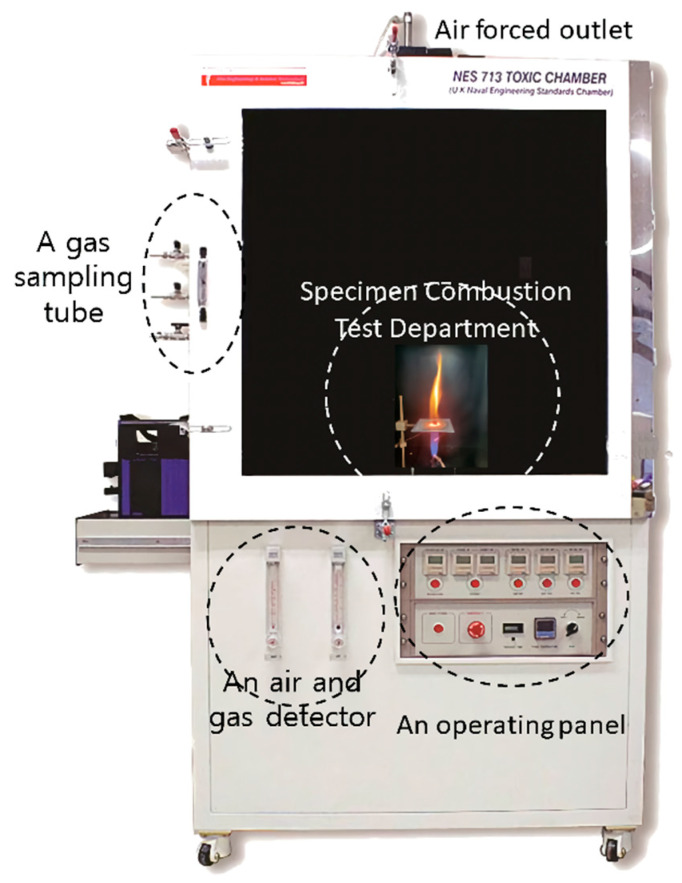
NES 713 testing equipment.

The test is performed using the apparatus shown in Figure 4. The NES 713 tester consists of a chamber, control panel, and sampling tube. The chamber contains a specimen support, a Bunsen burner to control the flame, a mixing fan to equalize the gas atmosphere in the chamber after combustion, a gas sampling ring tube, and a forced air extraction for internal ventilation after the end of the test. Each sample was cut to 1 ± 0.2 g and stored in a constant temperature and humidity chamber at 23 ± 1 °C and 50 ± 5% relative humidity for 24 h before testing. The prepared specimens were subjected to complete combustion for 2 min in a 100–120 mm high flame at a temperature of 1150 ± 25 °C by adjusting the ratio of oxygen and methane gas. After combustion, the mixing fan was operated and the gas was detected through the gas-detection tube using a gas detector.

Table 4 shows the concentration values of 13 gases (HF, HCl, HBr, SO_2_, H_2_S, HCN, HCHO, CH_2_CHCN, CO, CO_2_, NOX, NH_3_, C_6_H_5_OH) that are lethal after 30 min of exposure. Based on these values, the concentration of the gas (Cθ) is calculated using Equation (1). In the calibration experiment conducted for 2 min before this experiment, carbon dioxide (CO_2_) was detected at 4200 ppm, carbon monoxide (CO) at 10 ppm, and nitrogen oxide (NOX) at 1 ppm, and the calculated values were applied as calibration values. The values of 13 individual toxic gases calculated through this are converted to the toxicity index using Equation (2).

(1) $$C_{\theta }=\frac{C1\times 100\times V}{m}$$

where *C*1: The detected gas concentration (ppm); *V*: chamber volume (m^3^); *m*: test specimen mass (g).

(2) $$Toxicity\;Index=\sum_{i=1}^{n}\frac{C_{\theta 1}}{C_{f1}}+\frac{C_{\theta 2}}{C_{f2}}+\cdots +\frac{C_{\theta n}}{C_{fn}}$$

where 1, 2, 3 … *n*: converted gas concentration; *C_fn_*: lethal concentration for 30 min exposure (ppm) [23].

## 4. Result of Reaction to Fire Tests and Toxicity Test

### 4.1. Result of Reaction to Fire Tests

**Figure 5:**
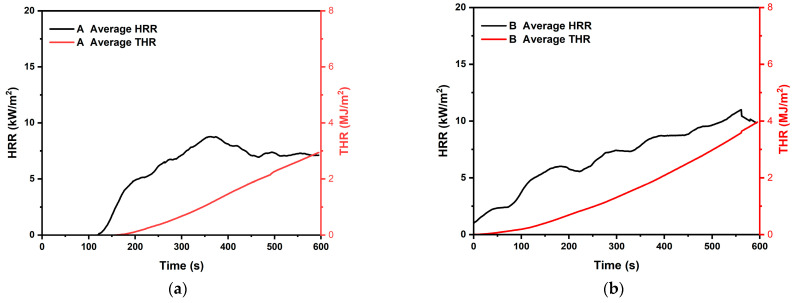

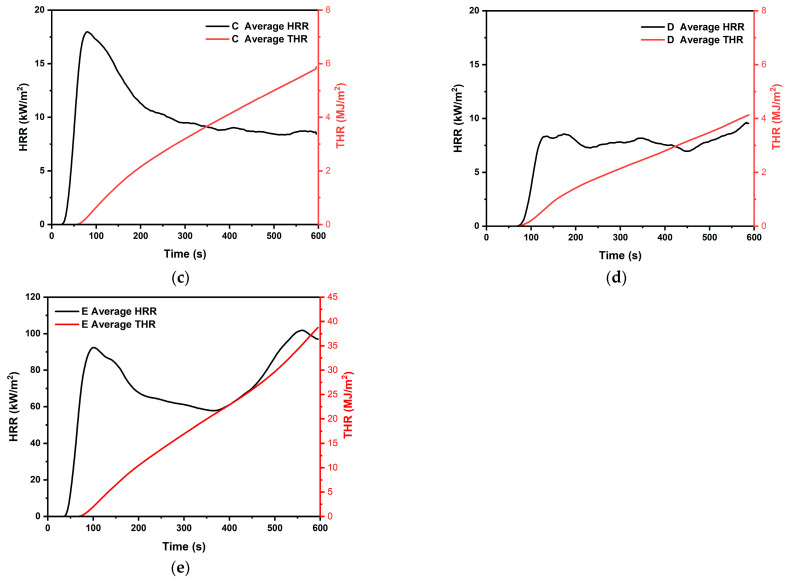
Specimen for ISO 5660-1 test THR and HRR. (**a**) Result for ISO 5660-1 Test piece A; (**b**) Result for ISO 5660-1 Test piece B; (**c**) Result for ISO 5660-1 Test piece C; (**d**) Result for ISO 5660-1 Test piece D; (**e**) Result for ISO 5660-1 Test piece E.

Figure 5 shows the results of the 10 min THR of the flat-board specimens. The average THR value of specimen A was the lowest at 2.9 MJ. The average THR value of each specimen was 3.9 MJ for specimen B, 5.8 MJ for specimen C, and 4.2 MJ for specimen D. In addition, all four specimens containing additives were not found to have a THR value of more than 8 MJ when tested for 10 min, which is the safety standard used in Republic of Korea and Japan.

In addition, the HRR values of specimens A, B, and D showed a moderate increase and maintenance after the initial ignition. However, specimen C showed a sharp increase in HRR after the initial ignition, followed by a decrease and maintenance within about 90 s. The average initial ignition time (IIT) of the specimens was found to be 70 s. For each specimen, the IIT was 116 s for specimen A, 31 s for specimen B, 42 s for specimen C, and 91 s for specimen D. The average initial ignition time was 70 s.

**Figure 6:**
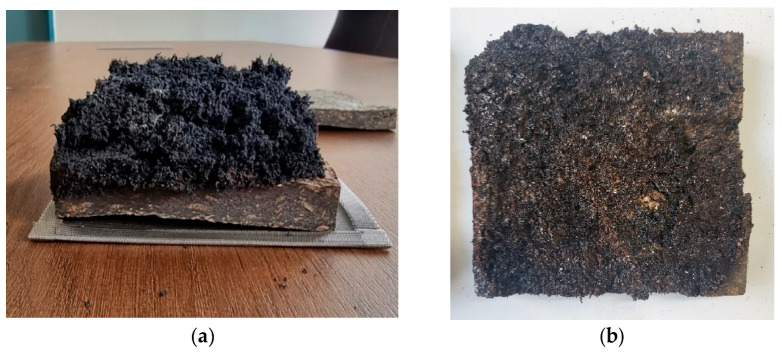
The appearance of the specimen after the end of the experiment. (**a**) Char barrier on post-experiment specimen “A”; (**b**) specimen “A” after removing the char barrier.

All the specimens used in this test showed THR values of 8 MJ or less for 10 min, which is rated as a Semi-Non-Combustible Material according to Korean standards. Also, Japan’s flame-retardant performance standard class 2 was confirmed. Figure 6 shows the appearance of the specimen after the end of the test. It was visually confirmed that there were no holes or cracks in the specimen through the carbonization surface. This shows the characteristics of the carbonized surface, which prevents the propagation of flame by forming a char film as the expanded graphite expands.

### 4.2. Result of Toxicity Test

The results of the toxicity evaluation showed that, out of a total of 13 gases, only 8 gases were detected except NH_3_, HCl, SO_2_, HBr, and HF. The detection results and toxicity indexes are shown in Table 5.

## 5. Discussion

### 5.1. Reliability of Specimens

To ensure that the specimens have the same fire characteristics, the standard deviation (*SD*) was calculated from the THR values of the given specimens. The formula is shown in Equation (3) below. Then, the *relative standard deviation* (*RSD*) value was calculated, which was utilized in the study of Ahn et al. Table 6 shows the mean, *SD*, and *RSD* values for the THR values obtained in this study.

(3) $$SD=\sqrt{\frac{1}{n}\sum_{i=1}^{n}{\left(x_{i}-M\right)}^{2}}$$

where *SD* = *standard deviation*; *n* = number of data; *M* = mean.

(4) $$Relative\;Standard\;Deviation\left(RSD\right)\left[\%\right]=\left(\frac{Standard\;Deviation}{Average}\right)\times 100$$

### 5.2. Toxicity Evaluation Results

The toxicity evaluation results were measured using a detector for a total of 13 gases. However, only 8 gases were detected: carbon dioxide (CO_2_), carbon monoxide (CO), hydrogen sulfide (H_2_S), formaldehyde (HCHO), acrylonitrile (CH_2_CHCN), nitrogen-oxides (NOX), phenol (C_6_H_5_OH), and hydrogen cyanide (HCN). Among them, the most toxic factors were CO (23.6%) for PF board, CO_2_ (28.4%) for compressed polystyrene foam, CO (53.1%) for MDF, and CO (64.3%) for hot-pressed panel board. Table 7 shows the toxicity index of NES-713 obtained in this study and the ratio of the toxicity index of each building material.

**Figure 7:**
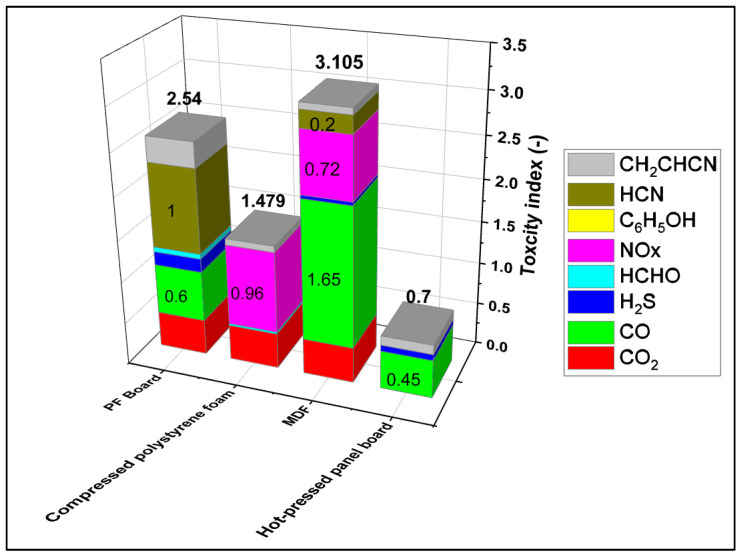
An evaluation results of toxicity index with building materials.

In addition, the evaluation of the overall toxicity index is shown in Figure 7. The hot-pressed panel board using waste paper was evaluated as having a lower toxicity index than conventional building materials. This shows that the safety of smoke toxicity in cases of fire is secured.

The toxicity index of the hot-pressed panel board is 0.7, and the safety against smoke toxicity is about 4.4 times higher than the toxicity index of MDF, which is about 3.1.

A study by Acuña et al. confirmed that the size of the expanded graphite particles creates a char barrier that reduces the heat flux, which also reduces the amount of smoke produced [24]. The study of Hwang et al. also showed that the use of ceramic binders reduced the size of the flame in ISO-11925 [25] (single-flame source test), showing that it prevents the spread of flame to the flame-retardant material [26]. Previous studies have confirmed that the most important factors affecting toxicity are related to the formation of a protective film and the amount of smoke generated, as in this study, and experimental results on smoke components have also demonstrated the effect of reducing the toxicity index.

This is believed to shorten the time that a loss of consciousness may last for fire victims due to smoke toxicity in the event of a fire and improve the evacuation time (A-SET) for fire victims, increasing the probability of survival.

## 6. Conclusions

The following conclusions were drawn from this study.

- First, the plate-like flame-retardant board produced through hot-pressing based on waste paper showed consistent flame-retardant properties and satisfied the Semi-Non-Combustible Material criteria of Korea’s Fire Performance Standards for Building Materials and those of fire retardancy class 2 for Japan’s Fire Performance Standard for Building Materials.
- Second, specimen A showed the lowest THR value and a delayed initial ignition time (50 Wt.% expanded graphite and 40 Wt.% ceramic binder), indicating flame-retardant performance.
- Third, the plate-like flame-retardant board produced in this experiment showed a low heat-release rate and toxicity value compared to conventional building materials, which suggests the possibility of ensuring an extension in the time required for human evacuation in cases of fire.

Through further research, we hope that the laboratory-scale hot-press manufacturing method in this study will be developed into a mass-production technology for building materials and that it will be used as a basis for the sustainable development of building materials in the field of construction in terms of utilizing waste paper.

## Figures and Tables

**Table 1:** Compositions of wet cellulose 3D printer filaments (Wt.%).

A Test Specimens Category	Waste Paper	Expanded Graphite	Ceramic Binder
A	100	50	40
B	100	50	30
C	100	40	40
D	100	40	30
E (no additive, cellulose-only)	100	0	0

**Table 2:** NES 713 appearance and components of test specimens.

Category	PF Board	Compressed Polystyrene Foam	MDF	Hot-Pressed Panel Board
Image	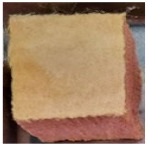	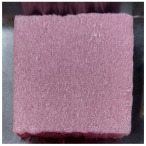	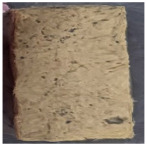	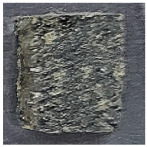
Main component	Phenolic resin	Extruded polystyrene foam	Medium-density wood fiber and binder	Waste paper, ceramic binder, expanded graphite

**Table 3:** A grade table of fire-resistant materials according to the fire characteristics of building materials in the Republic of Korea (dark gray) and Japan(Pale gray).

Standard	Korea Class	Japan Class	Heating Time (min)	Evaluation Criteria
ISO 5660-1 (Cone Calorimeter Method)	No criteria	Fire-retardancy class 1 (non-combustible)	20	- Total Heat Release (THR) is 8 MJ/m². - Max. Heat Radiant Rate (HRR) does not exceed 200 kW/m² for longer than 10 consecutive seconds. - There shall not be a crack that penetrates the sample, hole, or melting (for mixed-content materials, including melting and dissipation of all core materials) after heating for 10 min.
	Semi- non-combustible material	Fire-retardancy class 2 (limited combustion)	10	
	Fire-retardant material	Fire-retardancy class 3 (fire-retardant)	5	

**Table 4:** A lethal concentration for a 30-minute exposure (ppm).

Chemical Formula	A Lethal Concentration (for 30 min, ppm)	Chemical Formula	A Lethal Concentration (for 30 min, ppm)
CO₂	100,000	CH₂CHCN	400
CO	4000	NOx	250
H₂S	750	C_6_H_5_OH	250
NH₃	750	HCN	150
HCHO	500	HBr	150
HCl	500	HF	100
SO₂	400		

**Table 5:** Individual gas-detection levels and toxicity indices.

Category		Carbon Dioxide (CO_2_)	Carbon Monoxide (CO)	Hydrogen Sulfide (H_2_S)	Formaldehyde (HCHO)	Acrylonitrile (CH_2_CHCN)	Nitrogen-Oxides (NOX)	Phenol (C_6_H_5_OH)	Hydrogen Cyanide (HCN)	Toxicity Index
PF Board	Measurement value	5000	50	2	0.5	2	1	0	2.5	2.54
	$C_{\theta }$	0.42	0.6	0.16	0.06	0.3	0	0	1	
Compressed polystyrene foam	Measurement value	5000	10	0	0.2	0.5	5	0	0	1.479
	$C_{\theta }$	0.42	0	0	0.024	0.075	0.96	0	0	
MDF	Measurement value	5000	120	0.5	0	0.5	4	0	0.5	3.105
	$C_{\theta }$	0.42	1.65	0.04	0	0.075	0.72	0	0.2	
Hot-pressed panel board (specimen A)	Measurement value	4300	40	0.9	0	0.75	1	0	0	0.7
	$C_{\theta }$	0	0.45	0.072	0	0.1125	0	0	0	

**Table 6:** Validation of confidence intervals based on Total Heat Release (THR) values.

Specimen	Total Heat Release (MJ)	Average (kW/m^2^)	*Standard Deviation* (kW/m^2^)	*Relative Standard Deviation* (%)
A-1	2.877	2.94	0.083	2.818
A-2	2.89			
A-3	3.059			
B-1	4.262	3.92	0.247	6.299
B-2	3.687			
B-3	3.812			
C-1	5.67	5.8	0.099	1.699
C-2	5.836			
C-3	5.905			
D-1	5.67	4.15	0.319	7.681
D-2	5.836			
D-3	5.905			

**Table 7:** Relationship between Toxicity Index and content according to NES 713 testing.

Category		Carbon Dioxide (CO_2_)	Carbon Monoxide (CO)	Hydrogen Sulfide (H_2_S)	Formaldehyde (HCHO)	Acrylonitrile (CH_2_CHCN)	Nitrogen-Oxides (NOX)	Phenol (C_6_H_5_OH)	Hydrogen Cyanide (HCN)
PF Board	Toxicity index	0.42	0.6	0.16	0.06	0.3	0	0	1
	Ratio (%)	16.5	23.6	6.3	2.4	11.8	0.0	0.0	39.4
Compressed Polystyrene foam	Toxicity index	0.42	0	0	0.024	0.075	0.96	0	0
	Ratio (%)	28.4	0.0	0.0	1.6	5.1	64.9	0.0	0.0
MDF	Toxicity index	0.42	1.65	0.04	0	0.075	0.72	0	0.2
	Ratio (%)	13.5	53.1	1.3	0.0	2.4	23.2	0.0	6.4
Hot-pressed panel board (specimen A)	Toxicity index	0	0.45	0.072	0	0.1125	0	0	0
	Ratio (%)	0.0	64.3	10.3	0.0	16.1	0.0	0.0	0.0

## Data Availability

Data are contained within the article.
